# Development of a calculated panel reactive antibody calculator for the United Arab Emirates: a proof of concept study

**DOI:** 10.1038/s41598-023-34860-y

**Published:** 2023-05-25

**Authors:** Marion Alvares, Siddiq Anwar, Shahrukh K. Hashmi, Muhammad Badar Zaman, Ayeda Al Mahri, Christabelle Alvares, Layla Al Katheeri, Ananthanayagi Purushothaman, Mesele Emily Ralonya, Marie Glo Sangalang, Raysha Jannang, Abdulkadir Abdulle, Alyazia Al Qubaisi, Maitha Al Ahmed, Amar Hassan Khamis, Mohamed Al Seiari, Ali Al Obaidli, Zain Al Yafei, Gehad ElGhazali

**Affiliations:** 1 Transplant Immunology section, Sheikh Khalifa Medical City, Union71 - Purehealth, Abu Dhabi and College of Medicine and Health Sciences, United Arab Emirates University, Al Ain, United Arab Emirates; 2 Department of Medicine, Sheikh Shakbout Medical City, Abu Dhabi, United Arab Emirates; 3 Department of Internal Medicine, Mayo Clinic, Rochester, MN USA; 4 Clinical Affairs, Khalifa University, Abu Dhabi, United Arab Emirates; 5 Renal Transplant Department, Sheikh Khalifa Medical City, Abu Dhabi, United Arab Emirates; 6 Rochester Institute of Technology, New York, USA; 7 Mohamed Bin Rashed University of Medicine and Medical Sciences, Dubai, United Arab Emirates; 8 SEHA Kidney Care, Abu Dhabi, United Arab Emirates

**Keywords:** Immunology, Biomarkers, Health care, Medical research, Nephrology

## Abstract

Calculated panel reactive antibody (CPRA) is used to help increase sensitized patient’s access to transplantation. United Arab Emirates (UAE) has a diverse resident population hence we developed a UAE–CPRA calculator based on HLA antigen frequencies of the different ethnic groups that represent the UAE population. HLA antigen frequencies at serological split antigen level for HLA-A, -B, -C, -DRB1 and -DQB1 of 1002 healthy unrelated donors were performed. We subsequently compared the performance of the UAE CPRA calculator with the Organ Procurement and Transplantation Network (OPTN) and the Canadian CPRA calculators in 110 Kidney Transplant waitlist patients from January 2016 to December 2018. Lin’s concordance correlation coefficient showed a moderate agreement between the UAE and OPTN calculator (Rc = 0.949, 95% CI 0.929–0.963) and the UAE and Canadian calculators (Rc = 0.952, 95% CI 0.932–0.965). While there continued to be a moderate agreement (Rc = 0.937, UAE versus OPTN calculator) in the lower sensitized group, a poor agreement (Rc = 0.555, UAE versus OPTN calculator) was observed in the higher sensitized group. In this study, we provide a template for countries to develop their own population-specific CPRA calculator. Implementation of the CPRA algorithm based on HLA frequencies of the multi-ethnic UAE population will be more fitting to increase access to transplantation and improve transplant outcomes. Our study demonstrates that the CPRA calculators developed using the data from the western population had poor correlation in our higher sensitized patients disadvantaging them in potential organ allocations systems. We plan to further refine this calculator by using high resolution HLA typing to address the problem of a genetically diverse population.

## Introduction

Sensitization against preformed and de novo donor’s human leukocyte antigens (HLAs) can lead to hyperacute and antibody-mediated rejection in renal transplant recipients^1–3^. The percent of panel reactive antibodies (%PRA), i.e. % of this pool of donors to which a patient had reactive antibodies, is a major characteristic that defines the level of sensitization^4^. Essentially, greater PRA values indicate a higher percentage of likely cross-match incompatible donor, a lower probability of receiving a kidney transplant and a higher probability of antibody-mediated rejection unless adequately desensitized^2,5^. These HLA antibodies develop after exposure to blood transfusion(s), pregnancy, and previously failed graft(s)^6,7^. They are also known to have been produced by vaccinations and natural immunizing events, such as infections, protein ingestion, and allergen exposure^8,9^.

Solid-phase assays using single antigens (SA), e.g. Luminex bead assays developed using recombinant DNA technologies have increased the sensitivity and specificity to discern HLA-specific antibodies^10^. This improvement in sensitivity, accuracy, and precision has been an advancement in identifying unacceptable antigens and predicting virtual cross-matches especially in living-related transplants^11^.

Calculated PRA (CPRA) which was developed to standardize PRA reporting, is computed from HLA antigen frequencies representative of the organ donor population of a specific country or region^12^. CPRA is a highly useful metric, as it is based upon unacceptable HLA antigens to which the patient has been sensitized^13^. The precise data from solid‐phase antibody tests using the Luminex SA assays help to risk stratify and calculate the probability of the recipient matching with the potential donor pool. In the United Network for Organ Sharing (UNOS) data, the concordance of CPRA and PRA was high, with 90% of active renal candidates with a PRA 80% or higher having a CPRA in the same range^14^.

The United Arab Emirates (UAE) has a unique demographic with over 85% of the resident population being expatriate, with more than 200 nationalities living and working in the UAE^15^. The HLA population polymorphisms of the UAE would be different from other parts of the world that form the basis of well-known CPRA calculators^16^. Therefore, a more consistent and explicable method for measuring sensitization based on the UAE resident HLA antigens was much needed. In the UAE, the National Deceased Donor (DD) Program and the Kidney Paired Donation (KPD) program are both less than 5 years old. Virtual crossmatches and accurate CPRA estimates would be crucial in organ allocation prioritization for highly sensitized patients, as they will help avoid positive Complement dependent Crossmatches (CDC) and help define unacceptable HLA antigens to avoid delayed time in organ allocation^17,18^.

The research study aimed to develop a UAE-CPRA calculator by using HLA antigen frequencies of the different ethnicities that represent the resident UAE population. The Organ Procurement and Transplantation Network (OPTN) and the Canadian CPRA calculators are both easily available as web based tools that assign actual CPRA values to a transplant candidate based on the unacceptable antigens that are entered in the system. As the profile and ethnicity representation of donors used in this study are very different from the OPTN and the Canadian CPRA calculators we would expect differences, in CPRA data compared, across the online calculators. Therefore, using retrospective donor HLA typing data a UAE-CPRA calculator was first developed and it’s feasibility for implementation was studied by examining the antibody data of active sensitized waitlist patients in the UAE-CPRA calculator and comparing it with the Organ Procurement and Transplantation Network (OPTN)-CPRA and Canadian-CPRA online calculators.

## Materials and methods

### Data collection

This project was conducted in accordance with the ethical principles contained in the Declaration of Helsinki (2000). This study was approved by the Institutional Review Board/Research Ethics Committee of Sheikh Khalifa Medical City (SKMC), Abu Dhabi, UAE on 6th December 2020 (REC-06.12.2020 [RS-682]). The committee also waived the need for consent in view of the retrospective study design.

The Union71 Immunology and Histocompatibility laboratory at SKMC, Abu Dhabi, is the national reference laboratory supporting the four main solid organ transplant programs (Kidney, Liver, Lung, Heart) in the country. For this study HLA-A, -B, -C, -DRB1 and DQB1 typing of healthy renal and bone marrow donors (CPRA donor population) (n = 1002) registered in the database from Jan 2013 to Jun 2018 were retrospectively included in the current study (Table 1).

**Table 1:** Healthy donor distribution for the UAE-CPRA calculator.

Ethnic group/nationality	Number of donors included in the UAE-CPRA calculator
Emiratis	350
Other Arabs	352
South Asians	180
Southeast Asians	80
Other nationalities	40
Total	1002

Approximately one third of the donors were Emirati (indigenous population). The distribution of the other donor ethnicities was based on a similar distribution in the present donor population (Table 1). We ensured that the donor population was unrelated. All HLA typing was performed at our Immunology and Histocompatibility laboratory by PCR reverse sequence-specific oligonucleotide probe (PCR-RSSO) method (One Lambda Inc., Canoga Park, CA, USA) and were expressed as serological splits. Unacceptable antigen profiles of 110 sensitized potential Kidney Transplant waitlist patients (January 2016 to December 2019) with cumulative % Luminex SA > 10, tested for both HLA class I and II antibodies (Labscreen SA, One Lambda Inc., Canoga Park, CA, USA), were enrolled into this study. A cut-off of 1000 Mean Fluorescence Intensity (MFI) was used to determine the presence of HLA antibodies^19^. These patients were grouped as shown in Table 4. Three hundred and thirty one (n = 331) Kidney Transplants have been performed till September 2021 including 22 from deceased donors. The ethnic groups for the Recipients and Donors are listed in Tables 2 and 3.

**Table 2:** Recipients of SKMC transplant program until September 2021.

Ethnic group/nationality	No. of recipients
Emiratis	44
Other Arabs	119
South Asian	89
Southeast Asians	52
Other Nationalities	27
Total	331

**Table 3:** Ethnicity of living and deceased donors at SKMC transplant program until September 2021.

Living and deceased donor ethnic group/nationality	Total number of donors
Emiratis	32
Other Arabs	117
South Asian	101
Southeast Asians	54
Other Nationalities	27
Total	331

### Development of the UAE-CPRA calculator using HLA typing data

The calculated PRA by allele frequencies method was based on the formula used by the OPTN online calculator^20,21^. First, ethnic allele and haplotype frequencies for HLA-A, -B, -C, -DRB1, and -DQB1 were estimated by the expectation–maximization algorithm using the population genetics data software, Arlequin version. 3.5.2.2^22^. The UAE-CPRA calculator was developed based on these HLA antigen frequencies and the formula implemented by the OPTN online CPRA calculator [probability of a positive cross match = 1 − probability of a negative crossmatch = 1 − (1 − S1 + S2 − S3 + S4 − S5)^2^], where S1, S2, S3, S4, S5 represented the sum of all possible combinations of one, two, three, four and five loci haplotype frequencies respectively when the unacceptable antigens of a transplant candidate (TC) are entered in the calculator. The ethnic CPRA values generated for every ethnicity, using the above formula, are multiplied by ethnic weights and then totaled to give the final CPRA. The UAE-CPRA calculator was developed using the Java programming language.

### Data comparison with online CPRA calculators

Two sets of data comparisons were performed using CPRA calculators, a comparison of the HLA antigen frequencies between the CPRA calculators and a comparison of CPRA values for a set of sensitized patients derived from each of the three calculators. In the first dataset, HLA Class I and II antigens were separately entered into each CPRA calculator to give % antigen frequencies (AF). These AF were tabulated (Tables 5, 6) so that the differences in antigen frequencies could be compared. The table also includes the AF of the indigenous Emirati population in the current study. The second dataset was generated by entering the unacceptable antigens from the 110 sensitized patients individually into the UAE, OPTN, and Canadian CPRA calculators to generate CPRA scores for comparison. These scores were then grouped into two categories, a lower sensitized group (Group 1, %SA ≤ 50; n = 77) and a higher sensitized group (Group 2, %SA > 50, n = 33) as described in Table 4.

**Table 4:** Sensitized recipients’ categories used in the study.

Broad TC categories	Group	Cumulative single antigen (SA)	Number of patients
Lower sensitized n = 77	1	SA < 20%	21
		SA 20–50%	56
Higher sensitized n = 33	2	SA 51–80%	26
		SA > 80%	7

**Table 5:** HLA-A, -B, -C % antigen frequencies in the Emirati population and in the CPRA calculators used in this study.

HLA-A	Canadian	OPTN	UAE	EMIRATI current study	HLA-B	Canadian	OPTN	UAE	EMIRATI current study	HLA-C	Canadian	OPTN	UAE	EMIRATI current study
A1	28	24	16	8	B7	23	21	9	3	CW1	9	8	4	1
A2	47	48	38	20	B8	19	17	10	11	CW2	10	4	5	3
A3	25	22	14	6	B13	5	4	4	1	CW4	19	25	28	14
A11	12	10	17	10	B18	10	9	7	4	CW5	16	16	3	1
A23	4	7	5	2	B27	9	7	4	1	CW6	17	19	21	13
A24	19	17	24	9	B35	15	18	22	12	CW7	51	49	39	21
A26	6	5	12	8	B37	3	2	3	2	CW8	8	8	7	3
A29	8	7	4	1	B38	4	3	7	1	CW9	9	6	1	0
A30	5	8	12	6	B39	4	5	3	2	CW10	14	10	9	5
A31	7	5	6	3	B41	2	2	4	2	CW12	10	7	20	10
A32	6	5	12	8	B42	0	2	2	2	CW14	3	2	6	3
A33	3	5	10	6	B44	25	24	6	3	CW15	6	5	22	15
A34	1	1	0	1	B45	1	3	2	1	CW16	7	7	12	8
A68	7	11	12	9	B46	1	0	0	0	CW17	2	3	7	3
A69	0	0	1	0	B47	1	1	1	0	CW18	0	1	1	1
A74	0	2	1	0	B48	1	1	1	0					
					B49	4	3	4	1					
					B50	2	2	10	8					
					B51	12	10	24	16					
					B52	2	2	7	4					
					B53	1	4	4	2					
					B55	3	2	3	1					
					B56	1	1	0	0					
					B57	6	7	5	2					
					B58	3	4	8	6					
					B60	10	8	2	1					
					B61	4	4	11	8					
					B62	10	11	2	0					
					B63	1	1	3	1					
					B64	2	1	1	1					
					B65	6	4	2	2					
					B71	1	1	1	0					
					B72	1	2	2	1					
					B73	0	0	1	1					
					B75	1	0	2	0					
					B76	0	0	0	0					
					B77	0	0	0	0					
					B78	0	0	0	0					
					B81	0	1	0	0

**Table 6:** HLA-DRB1, -DQB1% antigen frequencies in the Emirati population and in the CPRA calculators used in this study.

HLA-DRB1	Canadian	OPTN	UAE	EMIRATI current study	HLA-DQB1	Canadian	OPTN	UAE	EMIRATI current study
DR1	16	19	10	4	DQ2	38	37	42	31
DR4	28	30	18	9	DQ4	7	10	6	2
DR7	25	22	22	12	DQ5	30	26	48	34
DR8	7	9	5	1	DQ6	41	37	34	15
DR9	3	3	2	0	DQ7	36	39	27	8
DR10	2	3	8	3	DQ8	18	24	13	7
DR11	18	19	19	7	DQ9	10	13	5	2
DR12	5	4	5	1					
DR13	21	22	18	7					
DR14	6	7	6	2					
DR15	26	26	26	10					
DR16	4	4	18	21					
DR17	22	18	25	20					
DR18	1	2	1	1					

### Data analysis

Agreement between the CPRA calculators, using data from the second dataset (CPRA scores for Groups 1 and 2), was analyzed using Bland–Altman plots. Concordance correlation was assessed by Lin’s concordance coefficient (Rc) using SPSS Version 21.0 (IBM SPSS Statistics for Windows, Version 21.0. Armonk, NY: IBM Corp). Lin’s coefficient was a reproducibility index that assessed the correlation between two readings that fall on a 45-degree line going through the origin^23^. An Rc value over 0.99 indicates an almost perfect agreement between the two methods, while a value of 0.95–0.99 means a substantial agreement. A moderate agreement is between 0.9 and 0.95 and a poor agreement is represented by a value < 0.90^23^.

## Results

### Antigen frequencies used for the UAE-CPRA calculator

We compared the antigen frequencies (AF) obtained in the three calculators to each other and to that of the Emirati population used in this study (Tables 5, 6). HLA-A2, -Cw7 were the most frequent HLA Class I antigens observed in all three CPRA calculators as well as in the Emirati population. However, HLA-B44 which had the highest frequency in the Canadian and OPTN CPRA calculators, was significantly less represented in the UAE-CPRA calculator and the Emirati group. On the other hand, HLA-B51 had the highest HLA-B AF in the UAE-CPRA calculator and the indigenous Emirati population. While HLA-DR4 and DR15 had the top 2 highest AF in the Canadian and OPTN-CPRA calculators, HLA-DR15 and DR17 and DR16 and DR17 were the most frequent HLA-DR antigens in the UAE-CPRA calculator and the Emirati population respectively. HLA-DQ5 had the highest HLA-DQ frequency in the UAE-CPRA calculator. It is also worth mentioning that HLA-Cw12 and Cw15 had significantly higher AF in the UAE-CPRA calculator as compared to the Canadian and OPTN-CPRA calculators. HLA-B76, B77, and B78 were not observed in any of the calculators, nor were they seen in the Emirati population.

### Haplotype frequencies used for the UAE-CPRA calculator

The most common Emirati Haplotypes and the other groups used in the UAE-CPRA calculator have been displayed in Tables 7, 8, 9 and 10^24^.

**Table 7:** Some common two locus HLA Haplotype Frequencies in the Emirati population as compared to other populations used in the study.

	Other Arabs	South Asian	Emiratis	Southeast Asians	Other Nationalities
A-B					
A2-B35	0.0095	0.0292	0.0341	0.0133	0.0125
A2-B50	0.0219	0.0138	0.0299	0	0
A2-B51	0.0461	0.0314	0.0568	0	0.025
A11-B61	0.0056	0.0105	0.0414	0	0.0125
A26-B8	0.0134	0.0303	0.0365	0.0062	0
B-DR					
B8-DR17	0.0316	0.0305	0.0929	0.00625	0.0375
B35-DR16	0.00426	0	0.0360	0	0.0375
B50-DR7	0.0506	0.0083	0.0482	0	0
B51-DR16	0.0170	0.0083	0.0518	0	0.05
B61-DR16	0.0033	0.0083	0.0525	0	0
DR-DQ					
DR4-DQ8	0.0723	0.0472	0.0670	0.0562	0.025
DR7-DQ2	0.1037	0.075	0.1055	0.0187	0.0625
DR15-DQ6	0.0852	0.1722	0.0812	0.0541	0.125
DR16-DQ5	0.0440	0.0444	0.2128	0	0.1125
DR17-DQ2	0.1234	0.0805	0.1957	0.0312	0.1

**Table 8:** Some common three locus HLA Haplotype Frequencies in Emirati population as compared to other populations used in the study.

	Other Arabs	South Asian	Emiratis	Southeast Asians	Other nationalities
A-B-C					
A2-B35-Cw4	0.0091	0.029658	0.0305	0	0.0125
A2-B50-Cw6	0.0246	0.0138	0.0281	0	0
A2-B51-Cw16	0.0237	0	0.0255	0	0
A11-B61-Cw15	0.0056	0.0064	0.0410	0.0062	0
A26-B8-Cw7	0.0139	0.0303	0.0353	0	0
B-Cw-DR					
B8-Cw7-DR17	0.0325	0.0305	0.0916	0.0062	0.0375
B35-Cw4-DR16	0.0028	0	0.0267	0	0
B50-Cw6-DR7	0.0466	0.0055	0.0431	0	0
B51-Cw16- DR16	0.0044	0	0.0259	0	0
B61-Cw15-DR16	0.0056	0.0083	0.0471	0	0.025

**Table 9:** Some common four locus HLA haplotype frequencies in Emirati population as compared to other populations used in the study.

	Other Arabs	South Asian	Emiratis	Southeast Asians	Other nationalities
A-B-Cw-DR					
A2-B50-Cw6-DR7	0.0191	0.0083	0.0277	0	0
A11-B61-Cw15-DR16	0	0	0.0273	0	0
A26-B8-Cw7-DR17	0.0014	0.0193	0.0341	0	0
A32-B51-Cw16-DR16	0.0014	0	0.0168	0	0
A68-B8-Cw7-DR17	0.0014	0.0027	0.0199	0	0
A-B-DR-DQ					
A2-B50-DR7-DQ2	0.0162	0	0.0294	0	0
A2-B51-DR16-DQ5	0.0056	0.0027	0.0238	0	0
A11-B61-DR16-DQ5	0.0028	0	0.0295	0	0
A26-B8-DR17-DQ2	0.0099	0.0194	0.0348	0	0
A68-B8-DR17-DQ2	0.0042	0	0.0194	0	0

**Table 10:** Some common five locus HLA haplotype frequencies in Emirati population as compared to other populations used in the study.

A-B-Cw-DR-DQ	Other Arabs	South Asian	Emiratis	Southeast Asians	Other nationalities
A2-B35-Cw4-DR16-DQ5	0	0	0.0170	0	0
A2-B50-Cw6-DR7-DQ2	0.0085	0.0083	0.0274	0	0
A11-B61-Cw15-DR16-DQ5	0.0028	0	0.0271	0	0
A26-B8-Cw7-DR17-DQ2	0.0099	0.0193	0.0327	0	0
A68-B8-Cw7-DR17-DQ2	0.0028	0	0.0199	0	0

### Correlation studies

Lin’s concordance correlation coefficient showed a moderate agreement between the UAE versus OPTN-CPRA calculators (repeatability coefficient-Rc = 0.949, 95% CI 0.929–0.963) and also between the UAE versus Canadian calculators (Rc = 0.951, 95% CI 0.932–0.965). The OPTN versus Canadian calculators showed a substantial agreement (Rc = 0.989) with each other. In broadly sensitized groups (%SA ≤ 50%, n = 77 and > 50%, n = 33), there continued to be a moderate agreement (Rc = 0.937, UAE vs. OPTN calculator) in the lower sensitized group but a poor agreement (Rc = 0.555, UAE vs. OPTN calculator) was observed in the higher sensitized group.

**Figure 1:**
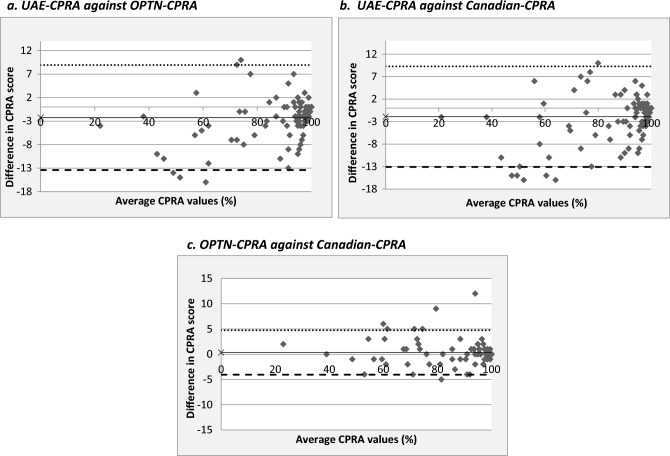
(**a**) UAE-CPRA against OPTN-CPRA. (**b**) UAE-CPRA against Canadian-CPRA. (**c**) OPTN-CPRA against Canadian-CPRA. *UAE CPRA* UAE calculated panel reactive antibody, *LOA* limits of agreement

Bland–Altman Plots described in Fig. 1a–c 1–3 with Table 11, showed that the limits of agreement (LOA) and standard deviation of difference (SD) between the UAE-OPTN calculators (Fig. 1a) and UAE-CANADIAN calculators (Fig. 1b) are similar. The highest agreement was observed to be between the OPTN-CANADIAN calculators (Fig. 1c).

**Table 11:** Statistical data representation for the Bland–Altman plots.

Statistical parameter	UAE vs OPTN CPRA	UAE vs CANADIAN CPRA	OPTN vs CANADIAN CPRA
	(Fig. 1a)	(Fig. 1b)	(Fig. 1c)
Average (vertical axis)	− 1.600	− 1.264	0.336
SD of difference	5.094	5.068	2.243
Correlation (r)	0.963	0.962	0.991
N	110	110	110
Lower LOA	− 11.584	− 11.196	− 4.061
Upper LOA	8.384	8.669	4.733

## Discussion

Antibody screening is routinely performed on patients waiting for solid organ transplants and measures HLA antibodies. In 1985 Zachary and Braun^20^ described a useful mathematical method for calculating a predictive value for transplantation known as CPRA. This CPRA mathematical formula gave rise to the OPTN-CPRA calculator being widely used by the UNOS to generate CPRA scores for transplant candidates that reflect the distribution of HLA antigens in the donor pool. In addition to improving the efficiency of organ allocation, the implementation of CPRA significantly improved the deceased donor transplant rates among candidates with CPRA greater than 98% in the United States^25^.

The Federal Law No 5 of 2016 allowing transplantation of human organs and tissues^26^ and the Ministerial decree No 550 in 2017 to enable confirmation of brain death resulting from complete loss of brain function^26^ has led to the establishment of a multi-organ transplant program in the UAE^27^.

The Union71 Immunology and Histocompatibility Laboratory at SKMC serves the UAE national transplant program and UAE national transplant committee with its function in waitlist management and organ allocation. It also serves the various transplant programs in the country with, HLA typing of potential donors and recipients, Luminex SA testing of potential waitlist recipients, virtual cross-matching, physical cross-matching and clinical consultations.

In 2005 our group published the frequencies of HLA-A, -B, -DR, and -DQ phenotypes in the United Arab Emirates resident population^28^. Since then the population demographics of UAE have changed having more nationalities immigrating to UAE.

As the transplant program in UAE is growing, it becomes evident that we need a customized CPRA calculator to ensure there is equity in organ allocation so no group of the population gets disenfranchised. At this early stage of the UAE deceased organ transplant journey, two observations have come to light, firstly there is a high polymorphism of HLA coupled with ethnographical differences between the UAE people and populations in the west which would require us to exercise due diligence in using readily available online CPRA calculators. Secondly, replacing % SA with CPRA as a sensitization measure would give a more uniform and consistent indicator of sensitization for all potential candidates across all transplant centers in the UAE^29^.

We developed our UAE-CPRA calculator on similar lines to the UNOS-CPRA calculator, enrolling five HLA locus unacceptable antigens (HLA-A, -B, -C, -DRB1, and -DQB1) from retrospective HLA data of 1002 healthy living donors^14^. The relatively small donor size is a major limitation of our calculator. Nevertheless, its intended use is to serve as a tool in the UAE where the ethnic HLA phenotypes are best represented and where western online CPRA calculators cannot be applied. We are aware that the frequency of some of the HLA antigens could be over or underestimated but the percentages of positive reactions may not be skewed by the sample size of the donor panel^18^. The preliminary UAE-CPRA calculator is the first version that was developed as proof of concept and it can easily be upscaled and adapted to have a more accurate representation of the countries’ donor population by introducing the HLA typing of new donors as more data becomes available. It may also be noted that Luminex SA assays allow for the identification of HLA-DQA1 and DP antibodies too, but they were not included in the CPRA calculator build as we do not usually HLA type or report these loci. However, all deceased donors are tested for HLA-A, -B, -C, -DRB1, -DRB3, 4, 5, -DQA1, -DQB1, -DPA1, and -DPB1. So in the event of strong anti HLA- DQA1 or DP antibodies being present, potential candidates may be excluded by a positive virtual crossmatch. Comprehensive HLA typing of donors is important for virtual crossmatch because sensitized patients have antibodies against the antigen products of all HLA loci. To overcome this limitation and to improve the accuracy of the UAE-CPRA calculator, the next version will include extended HLA typing to include HLA-DQA1, DPA1 and DPB1 of deceased donors, as in the current Canadian CPRA calculator{Tinckam, 2015 #29} and the NMDP-CPRA calculator. This will further improve equity, as many highly sensitized candidates also have antibodies to these loci^30,31^.

Common HLA Haplotypes in the UAE resident population, A26-B8-Cw7-DR17-DQ2 and A2-B50-Cw6-DR7-DQ2, are not prevalent in the west. It has been previously suggested that perhaps A1 in the A1-B8-Cw7-DR17-DQ2 (A*01:01-B*08:01-C*07:01-DRB1*03:01-DQB1*02:01, Ranked 1 in the USA NMDP European Caucasians) was replaced by A26 in the Emirati population^32^. On the other hand, A2-B7-CW7-DR15-DQ6 and A3-B7-CW7-DR15-DQ6 (A*02:01-B*07:02-C*07:02-DRB1*15:01-DQB1*06:02, Ranked 3 and A*03:01-B*07:02-C*07:02-DRB1*15:01-DQB1*06:02 Ranked 2 in USA NMDP European Caucasians; n = 1,242,890) were not observed at all in the Emirati group^33^. The phenotype and haplotype frequencies that were common among the Other Arabs, South Asians, and Emiratis were overall not so frequent in the Caucasian population. These Haplotype Frequencies (HF) differences have been reflected in the correlation studies performed here, wherein the UAE-CPRA calculator scores correlated poorly with the OPTN-CPRA calculator. On the other hand, western calculators showed a good correlation with each other.

Another important factor that may influence CPRA scores is the accurate determination of HLA antibodies that are relevant to transplantation. Most solid organ transplant programs list HLA to which there are complementary antibodies in the patient’s serum as unacceptable donor mismatches. The purpose of this is to facilitate organ allocation and avoid organ transplantation with increased immunological risk. The use of solid-phase antigen assays mitigates this of associated HLA being listed as unacceptable and which unnecessarily limits the patient’s access to transplantation or results in needless desensitization treatment.

The UAE-CPRA calculator includes a high AF of HLA-Cw12 and Cw15. Since false-positive Luminex Single Antigen Cw1, Cw12, and Cw15 beads are likely caused by denatured antigens, less sensitive Luminex PRA beads, having more native antigens, may need to be used for confirmation of these antibodies. Contrarily, a failure to identify relevant HLA antibodies (false negatives) may result in the transplantation of organs with which there is an unanticipated increased risk of immunological injury^34^. As our deceased donation program only started in 2018, our current conversion rates are low and during the study period the deceased donation was at 1.1 per million population (pmp)^35^. The population of UAE is very young and with a crude mortality rate of 1.51 deaths per 1000 population^36^.

The laboratories’ strategies towards testing and identification of HLA antibodies should include a clear knowledge of the patient’s history of sensitization, access to high-resolution HLA typing, knowledge of Epitope or CREG analysis, use of a combination of PRA and Single antigen (SA) testing when required and utilization of serum treatment protocols such as dilution, heat inactivation, addition of EDTA or treatment with DTT.

Since our laboratory currently tests all waitlisted candidates, we can deliver consistent results in both their HLA typing and HLA antibody identification for entry of unacceptable mismatches in the CPRA program.

The development of the UAE-CPRA calculator was at no extra cost and updating it as more donors are enrolled, will be considered to overcome the disadvantage of the small donor sample pool. It will be made available to the whole UAE transplant community to help improve equity and allow more rationale and optimize deceased donor allocation. The UAE-CPRA data analysis showed that about 80% of the 110 patients in the deceased organ waiting in this study were highly sensitized (CPRA > 80%). In addition, in the highly sensitized group, the UAE CPRA calculator indicated that the correlation with western CPRA calculators was poor, additionally justifying the implementation of a CPRA calculator which more closely reflects the resident population. These candidates would benefit from the allocation of additional points in the UAE organ allocation program which is currently under development.

As the UAE-CPRA calculator mainly uses HLA typing data from donors of the Indian subcontinent and the Middle East, it may serve as a model for developing an Indian or a Middle Eastern CPRA calculator. This study has several limitations among which are the small donor sample size, a single center study and unavailability of HLA-DQA1, DPA1 and DPB1 donor data in the CPRA calculation. Despite this, the development of the UAE-CPRA calculator using the resident UAE population is a more objective way of determining the sensitization status of waitlist patients.

In the era of precision medicine and the ever-evolving histocompatibility field, our future approach will consider including High-Resolution HLA typing of all solid organ recipients and donors and epitope-based HLA matching by using specialized computer programs that extend the repertoire of acceptable antigens^30,37–40^.

## Conclusion

A major challenge in our population is the diversity of the resident population which is mostly expatriate. As this population is constantly evolving, we will periodically evaluate the newly developed preliminary UAE-CPRA calculator at defined intervals to ensure we are accurately reflecting the resident population. The objective of this research study was to develop a UAE-CPRA calculator, which will serve our transplant waitlist population better by increasing access to transplant to sensitized individuals and reducing delays in deceased organ allocation. The implementation of the UAE-CPRA calculator will increase access to transplantation, improve transplant outcomes, will be useful in deceased organ allocation and setting up an equitable National KPD program. An accurate CPRA calculator will help UAE develop a national allocation that balances equity with efficiency to ensure no group of patients gets disadvantaged which is important for the transplant community to continue to have trust in a multicultural society like the UAE.

## Data Availability

The datasets generated and/or analyzed during the current study are not publicly available due to strict data residency laws regarding healthcare and especially genetic data but are available from the corresponding author after approval from the Department of Health, Abu Dhabi, UAE.
